# Temperature dependence of HCN channel kinetics: A systematic comparison across mammalian isoforms and species

**DOI:** 10.1113/EP093939

**Published:** 2026-08-30

**Authors:** Rajnish Ranjan, Emmanuelle Logette, Mirjia Herzog, Valerie Buchillier, Enrico Scantamburlo, Henry Markram

**Affiliations:** ^1^ Blue Brain Project Ecole Polytechnique Fédérale de Lausanne (EPFL) Geneva Switzerland; ^2^ Laboratory of Neural Microcircuitry Brain Mind Institute Ecole Polytechnique Fédérale de Lausanne (EPFL) Lausanne Switzerland

**Keywords:** automated patch clamp, CHO cell, electrophysiology, HCN channel, ion channel, kinetics, temperature, voltage clamp

## Abstract

Hyperpolarization‐activated, cyclic nucleotide‐gated (HCN) channels are important regulators of cardiac pacemaking and neuronal excitability, yet their temperature dependence has not been compared systematically across mammalian isoforms and species in standardized experimental conditions. Here, we performed a standardized electrophysiological characterization of mouse, rat and human HCN isoforms using stable CHO cell lines and automated patch‐clamp recordings across multiple temperatures and pharmacological conditions. Among the four isoforms, HCN3 did not generate detectable currents when expressed as a homomeric channel in the conditions tested, suggesting that additional factors might be required for functional activity. Comparative analysis of the remaining isoforms revealed a conserved kinetic hierarchy, with HCN1 exhibiting the fastest activation kinetics, HCN2 intermediate kinetics and HCN4 the slowest, and temperature accelerated channel gating across isoforms. Canonical modulation by cAMP and ZD7288 further showed that key regulatory features of HCN channels could be quantified reproducibly in identical experimental conditions. Together, these results provide a standardized comparative framework for interpreting HCN channel behaviour across physiological temperatures and species and establish a public reference dataset for future experimental and computational studies.

## Abstract (highlights)

- **What is the central question of this study?**
  How does temperature shape the kinetic properties of mammalian HCN channels across isoforms and species in standardized recording conditions?
- **What is the main finding and its importance?**
  Using automated patch‐clamp recordings, we show that temperature strongly accelerates the activation and deactivation kinetics of HCN channels while preserving the intrinsic kinetic hierarchy among isoforms. HCN3 is electrically silent in all tested conditions. cAMP and ZD7288 exhibit distinct isoform‐ and temperature‐dependent effects. These data, freely available through Channelpedia, provide a comparative framework for interpreting HCN function and improving electrophysiological experiments and computational models.

## 1 INTRODUCTION

Hyperpolarization‐activated, cyclic nucleotide‐gated (HCN) channels are key determinants of rhythmic electrical activity in both cardiac and neuronal systems. They belong to the superfamily of tetrameric voltage‐gated ion channels (Yu et al., 2005) and share structural features with both voltage‐gated potassium (Kv) channels and cyclic nucleotide‐gated (CNG) channels. In mammals, four HCN isoforms (HCN1–HCN4) have been identified (Ludwig et al., 1998; Santoro et al., 1998), each with distinct gating kinetics, cyclic nucleotide sensitivity and expression patterns that shape their physiological roles across excitable tissues (Chen et al., 2001; Moosmang et al., 2001; Robinson & Siegelbaum, 2003).

HCN channels are widely expressed in the heart and nervous system, where isoform‐specific and subcellular expression patterns further diversify their contribution to excitability (Kole et al., 2006; Lörincz et al., 2002). They also contribute to peripheral sensory signalling, including in dorsal root ganglion neurons, where HCN2 has been implicated in pain pathways (Emery et al., 2011). Functionally, HCN channels contribute to the hyperpolarization‐activated inward current, termed *I*_f_ in the heart and *I*_h_ in neurons, and thereby support spontaneous pacemaking, rhythmic firing, dendritic integration and rebound excitability (Biel et al., 2009; Postea & Biel, 2011; Shah et al., 2004).

HCN channel activity is regulated by both intrinsic gating properties and extrinsic modulatory factors. Among the best‐characterized modulators, cyclic nucleotides such as cAMP facilitate channel opening and modulate activation kinetics (Porro et al., 2024; Wainger et al., 2001). Conversely, ZD7288 has been widely used as a pharmacological inhibitor of HCN channels to probe the contribution of *I*_h_/*I*_f_ to cellular and tissue physiology (BoSmith et al., 1993; Wu et al., 2012). Although the biophysical and pharmacological properties of individual HCN isoforms have been described in many studies, most analyses have focused on selected isoforms, species or experimental conditions, making direct comparison across datasets difficult.

Temperature is a fundamental determinant of ion‐channel gating and is particularly important for HCN channels, whose relatively slow activation and deactivation kinetics strongly influence the timing of membrane excitability (Wu et al., 2025). Despite this, HCN channel properties have often been studied in variable thermal conditions, most commonly at room temperature rather than near‐physiological temperatures. This complicates the interpretation of published data, limits direct comparison across mammalian orthologues and constrains the use of HCN kinetic parameters in physiological and computational models. A standardized comparison of HCN channel kinetics across isoforms, species and temperatures is therefore needed to gain a better understanding of how thermal conditions shape functional channel behaviour.

Here, we performed a standardized electrophysiological characterization of mammalian HCN channels across isoforms, species, temperatures and canonical modulatory conditions. Using stable CHO cell lines expressing mouse, rat and human HCN channels and an automated patch‐clamp platform, we quantified channel behaviour in identical experimental conditions at 15, 25 and 35°C and examined modulation by cAMP and ZD7288. Our aim was to establish a comparative physiological framework for interpreting temperature‐dependent HCN channel kinetics across mammalian systems and to provide a reusable public reference dataset for future experimental and computational studies, rather than to resolve the molecular determinants of HCN temperature sensitivity directly.

## 2 MATERIALS AND METHODS

### 2.1 Ethical approval

No new animal experiments were performed for the electrophysiological recordings reported in this study, which were conducted entirely using stable heterologous cell lines. Animal tissue was used only at the start of the project for RNA isolation for ion channel cloning, as described previously (Ranjan et al., 2019). These procedures were carried out under animal authorization VD1550, approved by the Veterinary Authorities and the Cantonal Commission for Animal Experimentation of the Canton of Vaud, in accordance with Swiss animal protection law. Animals were housed with free access to food and water in standard institutional conditions, and tissue collection was performed in accordance with the approved protocol. No additional animals were used specifically for the present study.

### 2.2 RNA isolation and complementary DNA library synthesis

Total RNA was isolated from the whole brain, cerebellum or cerebral cortex of wild‐type 3‐month‐old male (C57BL6/J) mice or Wistar–Han rats. The brain tissue was collected in Qiazol**^®^** Lysis Reagent (Qiagen), ground with a plastic piston, sonicated for two or three pulses, followed by chloroform extraction and RNA purification using the RNeasy**^®^** Mini Kit (Qiagen), following the supplier's protocol. Five micrograms of total RNA was primed by an oligo‐dT primer and reverse transcribed using SuperScript**^®^** III Reverse Transcriptase (Invitrogen™‐Life Technologies) according to the manufacturer's instructions (final volume of 20 µL). For human gene amplification, PCR‐ready first strand human complementary DNA (cDNA) from the cerebellum, cortex and thalamus was purchased from BioChain Institute Inc. The RefSeq accession numbers, sequence information, and nucleotide modifications of the HCN genes used in this study are provided in Table A1.

### 2.3 HCN gene amplification

Primers specific for each HCN ion channel were designed using DNASTAR Lasergene SeqBuilder™ (Table A2). PCR amplification was performed using 2–3 µL of cDNA template with high‐fidelity Pwo SuperYield DNA Polymerase (Roche Life Science) in the presence of GC‐rich solution, using an Eppendorf thermal cycler.

For cloning, a two‐fragment assembly strategy was used for HCN4 and human HCN1 using the GENEART^®^ Seamless Cloning Kit (Invitrogen™, Life Technologies). Detailed PCR conditions for each ion channel are provided as datasheets on the Channelpedia website. Despite multiple attempts, rat HCN1 and human HCN1 could not be amplified from cDNA libraries and were therefore amplified from commercially obtained plasmids (pUC57, purified DNA, GenScript^®^; 4 µg input). Human HCN4 was amplified from a plasmid provided by the EPFL Gene Expression Core Facility.

Likewise, human HCN2 could not be amplified successfully from cDNA libraries or from the GenScript ORF plasmid. Consequently, amplification was performed using a pENTRY vector (GenScript^®^).

### 2.4 Cell line generation and maintenance

The Flp‐In™ CHO (Chinese Hamster Ovary; RRID: CVCL_U427) cell line was purchased from Invitrogen™‐Life Technologies. The Flp‐In™ CHO was converted in Flp‐In™ T‐Rex™ as previously described (Ranjan et al., 2019) to generate isogenic tetracycline‐inducible cell lines. For simplicity, Flp‐In™ T‐Rex™ is abbreviated as FT. The mouse, rat and human HCN cell lines were generated as previously described (Ranjan et al., 2019; cell lines library construction). Cell handling and maintenance for mentioned cell lines are described by Ranjan et al. (2019).

### 2.5 Induction of ion channel expression

Cells were plated and grown for 24 h in their respective culture medium, free of antibiotics. The number of cells and the type of culture vessels depended on the specific experiment. Ion channel expression was induced by adding 1 µg/µL tetracycline to the culture medium for 24 h. In the case of treatment, cells were treated with the indicated drug or compound in the culture medium at the time of tetracycline induction for 24 h.

### 2.6 Messenger RNA extraction and RT‐PCR

RNA was isolated using the RNeasy**^®^** Mini Kit (Qiagen), following the supplier's protocol. First‐strand cDNA was synthesized from 2 µg of total RNA using SuperScript**^®^** III Reverse Transcriptase and oligodT, according to the manufacturer's instructions, in a final volume of 20 µL. The expression of the targeted gene was then checked by PCR. Briefly, 1 µL of the cDNA product was used for PCR amplification (a 1:50 dilution of the cDNA was used for an overexpressed gene), using gene‐specific primers on a thermal cycler (SensiQuest) with 28 cycles of amplification. The housekeeping gene *gapdh* was used as the internal control.

### 2.7 Automated patch clamp

Automated patch‐clamp experiments were carried out as described before (Ranjan et al., 2019). In summary, a dedicated 2.5 m × 2.5 m temperature‐controlled room was used to record cells in whole‐cell patch clamp configuration on a Nanion NPC‐16 Patchliner Quattro (Nanion Technologies GmbH) fitted with EPC‐10 HEKA Quadro amplifiers (HEKA Electronik GmbH). Disposable borosilicate (Nanion NPC‐16) medium‐resistance (2–3 MΩ) glass chips were used for all recordings. The PatchControlHT software (Nanion Technologies GmbH) was used for the automation of patch‐clamp steps (cell capture, seal formation, whole‐cell access and washing, etc.). The PatchControlHT software internally used Patchmaster software (HEKA) for the data acquisition. The data were filtered with internal filter 1 (Bessel) at 10 kHz and filter 2 (I_Bessel) at 2.9 kHz of the EPC‐1 10 and digitized at 10–50 kHz configuration according to different voltage protocols. The extracellular solution (ECS) contained: 110 mM NaCl, 30 mM KCl, 0.5 mM MgCl_2_, 1.8 mM CaCl_2_, 5 mM Hepes; pH adjusted to 7.4 with NaOH, osmolarity ∼300 mosmol/L. The intracellular solution (ICS) contained: 70 mM KCl, 10 mM NaCl, 60 mM potassium fluoride, 0.5 mM MgCl_2_, 5 mM Hepes; pH adjusted to 7.4 with KOH, osmolarity 290 mosmol/L. The seal enhancer solution (SES) contained: 80 mM NaCl, 3 mM KCl, 10 mM MgCl_2_, 35 mM CaCl_2_, 10 mM Hepes (Na+salt); pH adjusted to 7.4 with HCl, osmolarity 298 mosmol/L. All solutions were filtered through a 0.2 µm filter (GP ExpressPLUS membrane, Millipore) and stored in 50 mL aliquots at +4°C.

### 2.8 Cell selection criteria for electrophysiology data

The first phase of cell selection for electrophysiology data involved evaluation of key recording parameters: voltage offset (V‐offset), whole‐cell seal resistance (post whole‐cell configuration), series resistance (R‐Series) and slow capacitance (C‐slow). The preliminary acceptance criteria were as follows: V‐offset < 45 mV, seal resistance > 200 MΩ, R‐Series < 15 MΩ and C‐slow < 35 pF.

These criteria effectively eliminated the majority of poor‐quality recordings. However, in some cases, cells that met all these criteria still exhibited unstable membrane currents, which could not be identified solely through standard electrophysiological metrics. Such cells were reviewed manually and excluded from the final analysis.

### 2.9 Stimulation protocols

#### 2.9.1 Resting potential

The resting potential of each cell was recorded in current‐clamp mode by applying zero current for 5 s. The protocol was repeated three times with 6 s intervals after each repetition.

For all voltage‐clamp protocols except action potential (AP), cells were held at a holding potential of −40 mV and had an identical beginning (first 100 ms) and end. The first 100 ms were divided into three parts: 40 ms at holding potential (−40 mV), 10 ms at −50 mV and the remaining 50 ms at holding potential (−40 mV). The last 100 ms were always held at the holding potential (−40 mV).

#### 2.9.2 Activation

**FIGURE A1:**
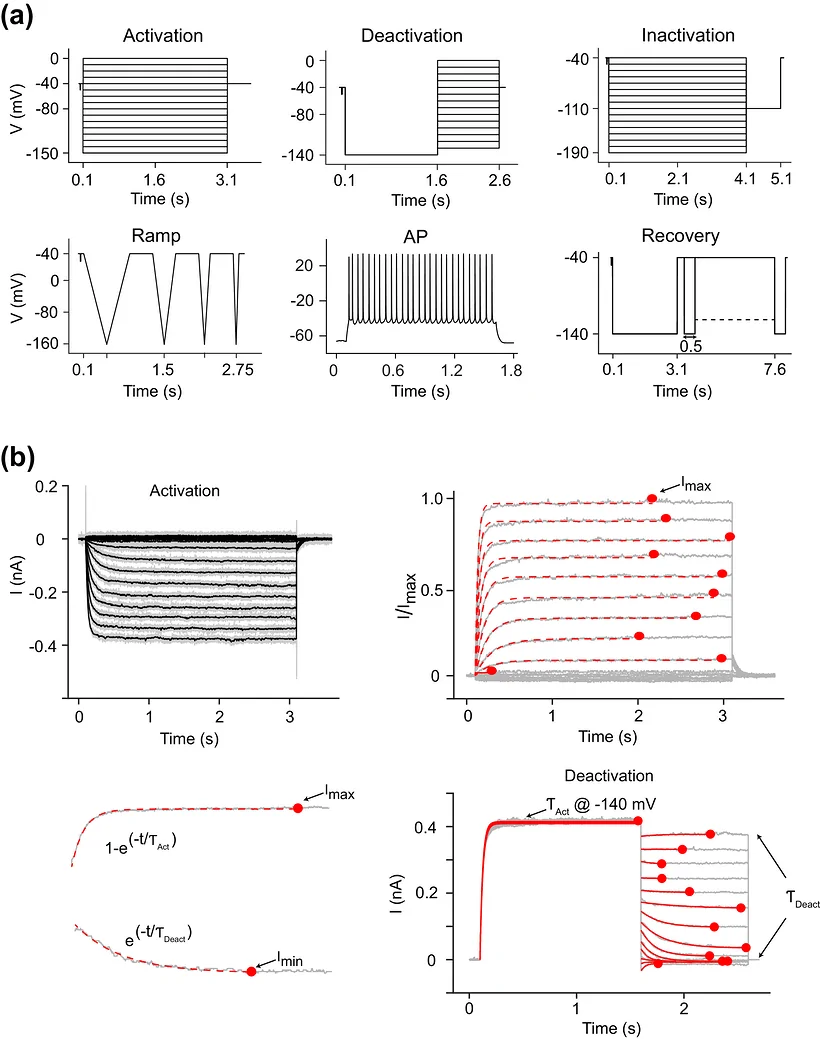
Electrophysiological protocols and feature extraction workflow for HCN channels (a) Voltage‐clamp protocols used to extract kinetic parameters of mouse HCN channels. Protocols included activation, deactivation, inactivation, ramp stimulation, action potential (AP) waveform stimulation, and recovery protocols. Hyperpolarizing voltage steps were used to evoke HCN currents and characterize voltage‐dependent gating properties. (b) Schematic illustration of the feature extraction workflow used to quantify activation and deactivation kinetics. For activation analysis, peak current amplitudes (Imax) were identified for each trace, normalized to the maximal current within the protocol, and used to construct normalized current‐voltage relationships. Activation time constants (τAct) were obtained by fitting the rising phase of the current with a single‐exponential function. For deactivation analysis, currents were first activated at −140 mV, and deactivation time constants (τDeact) were determined by fitting the decay phase of tail currents with a single‐exponential function.

To probe the steady‐state activation kinetics, a conventional step voltage protocol containing 16 sweeps was applied, starting from 0 mV, to −150 mV in 10 mV step decrements; each sweep duration was 3 s, and after each sweep the cells were held at the holding potential (−40 mV) for 500 ms and the inter‐sweep interval was 5 s (Figure A1a).

#### 2.9.3 Deactivation

To probe the steady‐state deactivation kinetics, a two‐pulse protocol with 14 sweeps was designed. The first pulse, of 1.5 s duration at −140 mV, was applied to induce opening of the channel, and the second pulse, of 1 s duration, with voltage steps from 0 mV to −130 mV in 10 mV decrements, was applied to measure the deactivation kinetics that occurred as the voltage was changed from one level to another (Figure A1a).

#### 2.9.4 Inactivation

To study the steady‐state voltage dependence of inactivation, a two‐pulse protocol with 16 sweeps was designed. The conditioning pulse, of 4 s duration, with voltage steps from −40 to −190 mV in 10 mV step decrements, was applied to observe maximum inactivation. The following brief test pulse, 1 s in duration, at a fixed voltage of −120 mV, was applied to measure inactivation induced by the conditioning pulse (Figure A1a).

#### 2.9.5 Recovery

To study the dynamics of recovery from inactivation, a two‐pulse protocol with 16 sweeps was designed. The first pulse, of duration 3 s at −140 mV, was applied to induce inactivation; then a test pulse of duration 500 ms at −140 mV was applied after holding the cell at a recovery potential of −40 mV for variable intervals, from 25 ms to 4.525 s in steps of 300 ms (Figure A1a).

#### 2.9.6 Ramp

To assess the response of HCN channels to subthreshold voltage fluctuations, a series of four triangular voltage pulses between −40 and −160 mV was applied, with varying duration (400, 200, 100 and 50 ms) and with a 400 ms interval between each pulse. A total of five sweeps, with an inter‐sweep interval of 5 s, was applied for this protocol (Figure A1a).

#### 2.9.7 Action potential

A 1.8‐s‐long regular spiking action potential voltage protocol was applied to mimic physiological stimuli. The train of action potentials at 18 Hz was obtained from somatic whole‐cell current‐clamp recordings from layer 2/3 pyramidal neurons at 34 ± 1°C in rat somatosensory cortex slices from animals at postnatal day 14 (Figure A1a).

#### 2.9.8 Drug

To probe the effect of a drug (e.g. ZD7288), a continuous pulse of 700‐ms‐long hyperpolarizing step voltage pulses of amplitude −140 mV was applied with an interval of 30 s.

### 2.10 Feature extraction

For each recorded cell, kinetic features were extracted from every protocol and from each protocol repetition using custom MATLAB scripts. All analyses, including parameter estimation and curve fitting, were performed in MATLAB using the ‘fminsearch’ function.

Before feature extraction, electrophysiological traces were preprocessed by cleaning, denoising, smoothing and inverting where required to facilitate automated analysis, as illustrated in Figure A1b.

For the activation protocol, traces between 100.03 and 3099 ms were analysed. For each trace, the maximal current value (*I*_max_) was identified using a moving median filter with a window size of 200 points. These peak values, indicated by red dots in Figure A1b, were then normalized to the maximum current recorded within the protocol. The normalized peak current at each command voltage was used to generate the current–voltage (*I–V*) relationship. The normalized peak current‐voltage relationship was then fitted with a sigmoidal (Boltzmann) function, and the half‐activation voltage ($V_{1/2}$) was defined as the membrane potential at which the fitted normalized activation curve reached 50% of maximal activation. Activation kinetics were quantified by fitting a single‐exponential function, 1 − exp(−*t*/τ_Act_), from 100.03 ms until the time of peak current for each trace. The resulting activation time constant (τ_Act_) was plotted as a function of command voltage to generate τ_Act_–*V* relationships.

For the deactivation protocol, traces were preprocessed in the same manner. Initially, activation at −140 mV was quantified by fitting the rising current between 100.03 and 1599.8 ms with a single‐exponential function to extract τ_Act_ at −140 mV. Deactivation kinetics were then analysed over the interval from 1600.03 to 2599.8 ms. Within this window, the minimum current value (*I*_min_) was identified using the same moving median approach, and the decay phase was fitted with a single‐exponential function, exp(−*t*/τ_Deact_), to obtain the deactivation time constant (τ_Deact_).

## 3 RESULTS

### 3.1 Standardized electrophysiological characterization identifies HCN3 as an electrically silent channel

**FIGURE 1:**
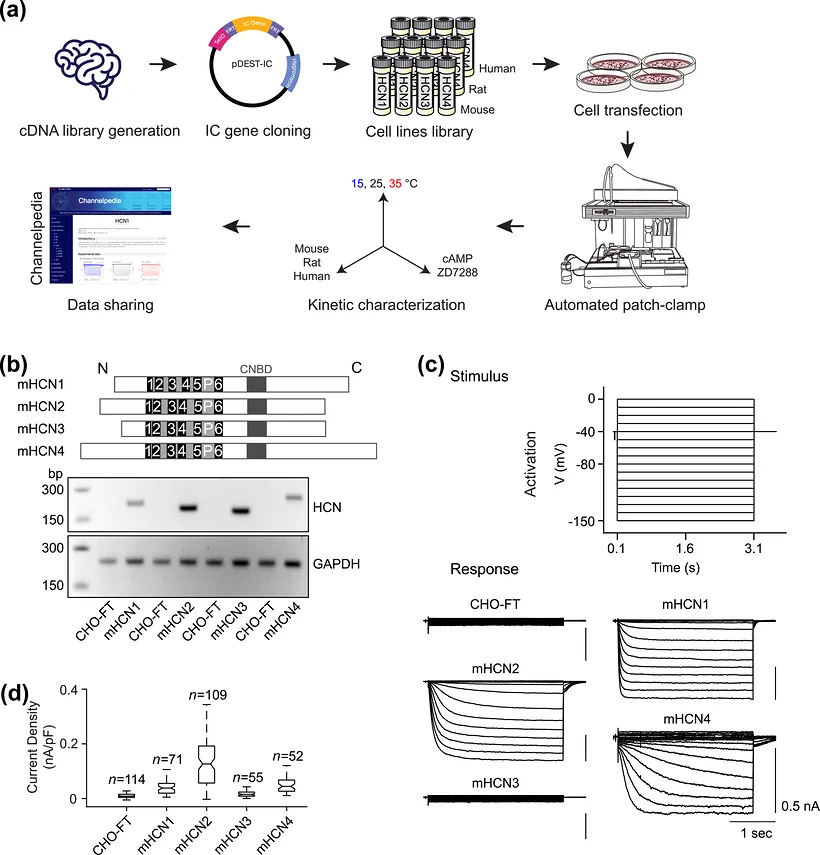
Standardized experimental workflow for large‐scale kinetic characterization of HCN channels. (a) Overview of the experimental workflow used for standardized kinetic characterization of HCN channels. Complementary DNA libraries were generated, and individual ion channel (IC) genes encoding HCN1–HCN4 from mouse, rat and human were cloned into the pDEST‐IC mammalian expression vector. Stable HCN‐expressing cell lines were generated by transfecting each expression vector into the host CHO‐FT cell line for functional characterization. Electrophysiological recordings were performed using an automated patch‐clamp system in a dedicated temperature‐controlled environment in multiple experimental conditions, including different temperatures (15, 25 and 35°C), species orthologues (mouse, rat and human) and pharmacological modulators (cAMP and ZD7288). The resulting electrophysiology datasets were integrated into the Channelpedia database for data sharing and analysis. (b) Schematic representation of the structural organization of mouse HCN channels showing the six transmembrane segments (1–6) and the conserved cyclic nucleotide‐binding domain (CNBD). Expression of individual *mHCN1–mHCN4* transcripts in CHO‐FT cells was verified by RT‐PCR. *GAPDH* mRNA expression was used as a control. (c) Voltage‐clamp stimulus protocol and representative current responses used to assess activation kinetics of the HCN channel. Robust inward currents were observed for mHCN1, mHCN2 and mHCN4, whereas mHCN3, similar to control CHO‐FT cells, did not exhibit detectable HCN‐mediated currents. (d) Summary of current density measured in control CHO‐FT cells (*n* = 114) and in HCN cell lines expressing mHCN1 (*n* = 71), mHCN2 (*n* = 109), mHCN3 (*n* = 55) and mHCN4 (*n* = 52). Current density in mHCN3‐expressing cells was not significantly different from CHO‐FT cells but was significantly lower than in mHCN1, mHCN2 and mHCN4. Box plots show the median and interquartile range; notches indicate the approximate confidence interval around the median, and whiskers extend to the most extreme data points within 1.5 times the interquartile range. Outlier symbols are omitted for clarity. Sample sizes are indicated in the panel; *n* denotes the number of individual quality‐controlled recorded cells included in each analysis. Exact *P*‐values for the statistical comparisons are provided in Table A3.

**FIGURE A2:**
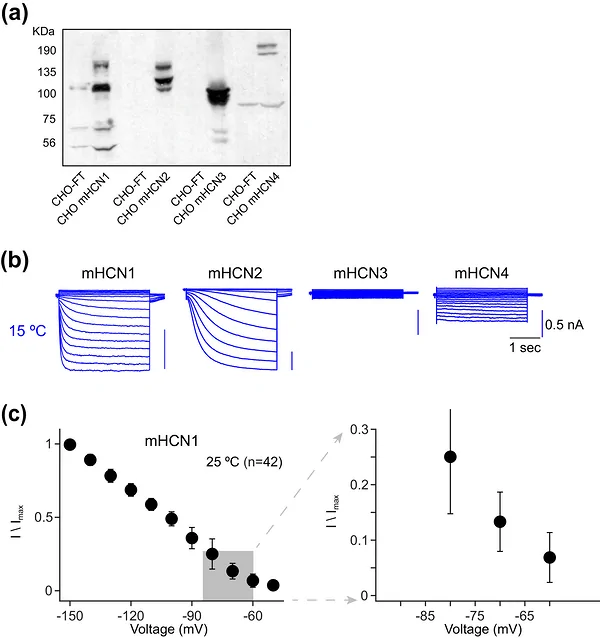
Mouse HCN channel expression and temperature‐dependent electrophysiological properties (a) Representative immunoblot showing expression of mouse HCN1, HCN2, HCN3, and HCN4 channels in stable CHO cell lines. Lysates from parental CHO cells (CHO‐FT) and CHO cells expressing individual HCN isoforms were probed to confirm expression at the expected molecular weights. (b) Representative whole‐cell current traces recorded from CHO cells expressing mouse HCN1, HCN2, HCN3, and HCN4 channels at 15°C during hyperpolarizing voltage steps. Robust inward currents were observed for HCN1, HCN2, and HCN4, whereas HCN3 remained electrically silent under these conditions. (c) Left panel, normalized current‐voltage (I/Imax) relationship for mouse HCN1 at 25°C, as shown in Figure 2B; right panel, expanded view of the subthreshold voltage range (−85 to −65 mV). The expanded view illustrates that mHCN1 already exhibits measurable activation within this range, corresponding to approximately 10–20% of the maximal normalized current.

To establish a standardized framework for comparative analysis of HCN channel kinetics, stable CHO cell lines expressing mouse, rat and human HCN isoforms were generated and examined using an automated patch‐clamp platform in temperature‐controlled conditions (Figure 1a). Gene expression of the mouse HCN cell lines was confirmed by RT‐PCR prior to electrophysiological recordings (Figure 1b), and protein expression was verified by western blot analysis (Figure A2a). Recordings were performed at 15, 25 and 35°C to assess temperature‐dependent channel behaviour in identical experimental conditions.

To probe channel kinetics comprehensively, six voltage‐clamp protocols were applied: activation, deactivation, inactivation, ramp, action potential (AP) and recovery (Figure A1a). In total, 7123 cells were recorded, of which 5035 met the predefined quality‐control criteria for qualitative analysis and 2704 met the stricter criteria for quantitative analysis. Consistent with the gating properties of HCN channels, the most informative features in the present study were derived primarily from activation and deactivation protocols.

A prominent finding from this systematic characterization was the absence of detectable current in cells expressing mouse HCN3. Representative activation traces showed robust hyperpolarization‐activated inward currents for mHCN1, mHCN2 and mHCN4, whereas responses recorded from mHCN3‐expressing cells were indistinguishable from those of parental CHO‐FT host cells (Figure 1c). Consistent with this observation, the overall group effect on current density was significant (Kruskal–Wallis test, *P* < 0.0001). *Post hoc* pairwise comparisons showed that current density in mHCN3‐expressing cells was not significantly different from CHO‐FT cells (*P* = 0.115) but was significantly lower than in mHCN1 (*P* = 0.000547), mHCN2 (*P* < 0.0001) and mHCN4 (*P* < 0.0001) (Figure 1d).

The same pattern was observed for rat and human HCN3, which likewise failed to generate detectable currents in the conditions tested. In addition, mouse HCN3 expressed in an independent heterologous expression system (HEK cells) did not produce measurable currents (data not shown). Together, these results indicate that HCN3 does not generate detectable homomeric currents in the heterologous systems examined here, suggesting that additional cellular factors might be required for functional activity in these experimental conditions.

### 3.2 Temperature accelerates mouse HCN channel gating and sharpens isoform‐specific kinetic separation

**FIGURE 2:**
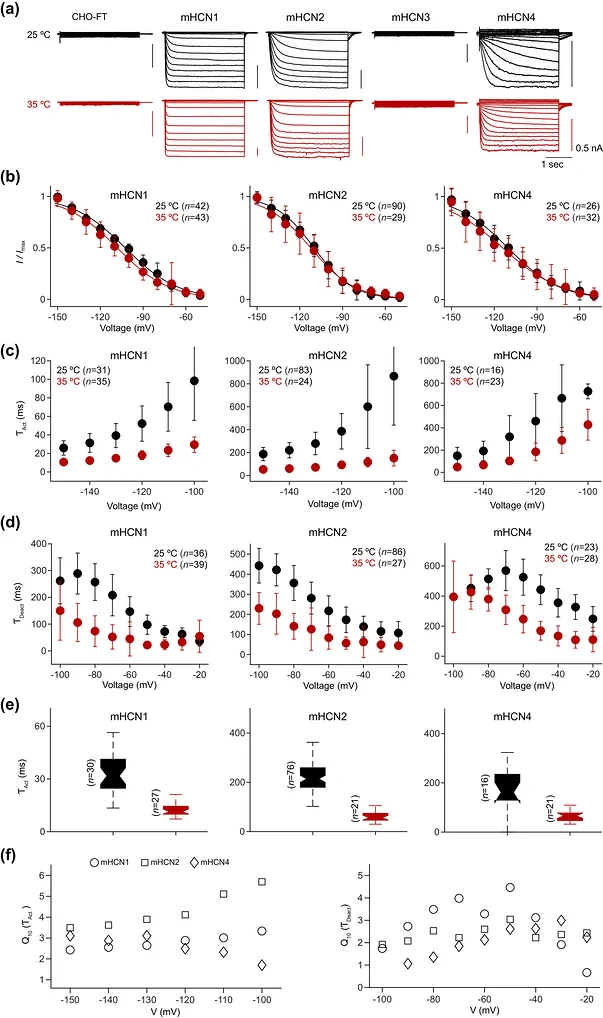
Temperature‐dependent modulation of mouse HCN channel kinetics. (a) Representative whole‐cell current traces in response to the activation protocol, recorded from CHO‐FT cells and cells expressing mouse HCN isoforms (mHCN1–mHCN4) at 25 (black) and 35°C (red) during hyperpolarizing voltage steps. Robust inward currents were observed for mHCN1, mHCN2 and mHCN4, whereas mHCN3, similar to control CHO‐FT cells, did not produce detectable HCN‐mediated currents. (b) Normalized activation curves (*I*/*I*_max_–*V*) for mHCN1, mHCN2 and mHCN4 at 25 and 35°C. Increasing temperature produced a significant leftward shift in the voltage dependence of activation for mHCN1 (∼6 mV; *P* < 0.0001). Smaller leftward shifts of ∼2–3 mV were observed for mHCN2 (*P* = 0.118) and mHCN4 (*P* = 0.214). The number of cells analysed is indicated in the panels. Symbols represent the mean ± SD. (c) Voltage dependence of activation time constants (τ_Act_) for mHCN1, mHCN2 and mHCN4 at 25 and 35°C. Activation kinetics were obtained by fitting a single‐exponential function from the onset of the hyperpolarizing stimulus to the time of maximal current. Increasing temperature markedly accelerated activation across all channels. Consistent with known isoform‐specific kinetics, mHCN1 exhibited the fastest activation, mHCN4 the slowest, and mHCN2 intermediate kinetics. Symbols represent the mean ± SD. (d) Voltage dependence of deactivation time constants (τ_Deact_) for mHCN1, mHCN2 and mHCN4 at 25 and 35°C. Increasing temperature substantially accelerated deactivation, leading to faster channel closure across the tested voltages. Symbols represent the mean ± SD. (e) Comparison of activation time constants (τ_Act_) measured at −140 mV for mHCN1, mHCN2 and mHCN4 at 25 and 35°C. Activation kinetics were significantly faster at 35 than at 25°C for all three isoforms (*P* < 0.0001 for each comparison). Box plots show the median and interquartile range; notches indicate the approximate confidence interval around the median, and whiskers extend to the most extreme data points within 1.5 times the interquartile range. (f) Temperature coefficients (*Q*_10_) calculated for activation and deactivation kinetics across voltages. Activation *Q*_10_ values indicate pronounced temperature sensitivity for HCN2 compared with HCN1 and HCN4, whereas deactivation *Q*_10_ values demonstrate distinct isoform‐dependent kinetic responses. *Q*_10_ values in (f) were derived from the activation and deactivation data (shown in c and d, respectively) and therefore reflect the same number of analysed cells. Sample sizes are indicated in the panels; *n* denotes the number of individual quality‐controlled recorded cells included in each analysis. Exact *P*‐values for the statistical comparisons are provided in Table A3.

A total of 1115 recordings were obtained from mouse HCN‐expressing CHO cells during kinetic characterization experiments, of which 858 passed predefined quality‐control criteria and were included in the final analysis. Representative activation traces recorded at 25 and 35°C are shown in Figure 2a, and recordings at 15°C are shown in Figure A2b. mHCN3 remained electrically silent at all temperatures tested, with responses indistinguishable from parental CHO‐FT cells. Because 15°C is well below both room temperature and physiological conditions, subsequent comparative kinetic analyses focused primarily on mHCN1, mHCN2 and mHCN4 recorded at 25 and 35°C.

To assess the effect of temperature on voltage‐dependent activation, normalized current–voltage (*I*/*I*_max_–*V*) relationships were derived from activation protocols. Increasing the temperature from 25 to 35°C shifted the activation curves of mHCN1 leftwards by ∼6 mV, indicating activation at more hyperpolarized potentials at the higher temperature (*P* < 0.0001). Smaller leftward shifts of ∼2–3 mV were observed for mHCN2 and mHCN4, but these differences were not statistically significant (mHCN2, *P* = 0.118; mHCN4, *P* = 0.214; Figure 2b; Table A3).

Activation kinetics were quantified by fitting the rising phase of the current with a single‐exponential function to obtain τ_Act_, as illustrated in Figure A1b. Across voltages, mHCN1 showed the fastest activation kinetics at both temperatures. However, the relative ordering of mHCN2 and mHCN4 depended on temperature. At 25°C, activation time constants of mHCN2 were comparable to, and at some voltages greater than, those of mHCN4, indicating that the previously described HCN1–HCN2–HCN4 kinetic hierarchy (Moosmang et al., 2001) was not cleanly resolved in these conditions. In contrast, at 35°C, this hierarchy became more distinct, with mHCN1 remaining the fastest (τ_Act_ ≈ 12–35 ms), mHCN2 intermediate (τ_Act_ ≈ 60–200 ms) and mHCN4 the slowest (τ_Act_ ≈ 60–600 ms)‐activating isoform across the tested voltage range. Thus, increasing temperature not only accelerated activation kinetics overall but also sharpened kinetic separation among the functional mouse HCN isoforms.

Temperature sensitivity of activation kinetics was further quantified using the *Q*_10_ coefficient (Ranjan et al., 2019). Among the channels tested, mHCN2 exhibited the strongest temperature dependence, with a mean *Q*_10_ of ∼4.3, whereas mHCN1 showed moderate sensitivity (*Q*_10_ ≈ 2.8) and mHCN4 exhibited the lowest temperature sensitivity (*Q*_10_ ≈ 2.6; Figure 2f).

Temperature also strongly affected deactivation kinetics. Deactivation time constants decreased markedly at 35 compared with 25°C, indicating faster channel closing at higher temperature across all three HCN isoforms (Figure 2d). The temperature coefficient for deactivation (*Q*_10_) showed voltage dependence, with values ranging from ∼1 to 5 across the tested voltage range (Figure 2f, right panel).

To compare the temperature effects across isoforms directly, τ_Act_ values measured at −140 mV were summarized for each channel (Figure 2e). At this voltage, activation kinetics were significantly faster at 35 than at 25°C for mHCN1 (*P* < 0.0001), mHCN2 (*P* < 0.0001) and mHCN4 (*P* < 0.0001; Table A3).

These comparisons highlight two key features of mouse HCN channel gating: (1) increasing temperature substantially accelerates activation across all functional isoforms; and (2) the canonical kinetic ordering of HCN1 fastest, HCN2 intermediate and HCN4 slowest is most clearly evident at 35°C, whereas at 25°C mHCN2 and mHCN4 are less distinctly separated.

### 3.3 Temperature‐dependent kinetic modulation of HCN channels by cAMP

**FIGURE A3:**
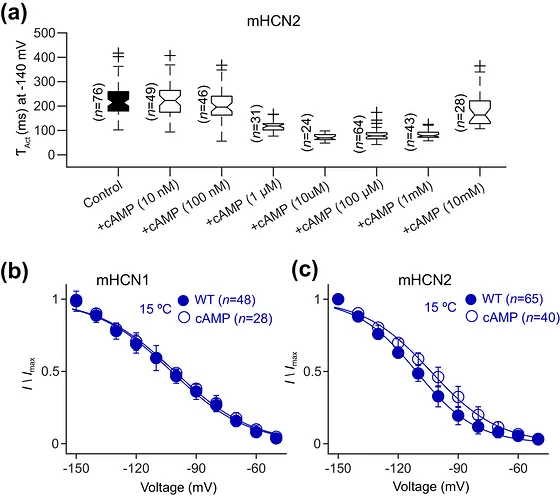
Concentration‐dependent and temperature‐specific modulation of mouse HCN channel activation by intracellular cAMP. (a) Concentration‐dependent effects of intracellular cAMP on mHCN2 activation kinetics. Activation time constants (τ_Act_) measured at −140 mV are shown for intracellular cAMP concentrations ranging from 10 nM to 10 mM. Increasing cAMP progressively accelerated channel activation up to the 10–100 µM range, with little additional effect at higher concentrations. Box plots show the distribution of τ_Act_ values across cells. (b) Normalized activation curves (*I*/*I*_max_) for mHCN1 recorded at 15°C in control conditions (WT) and in the presence of 100 µM cAMP. In these conditions, cAMP produced little or no detectable shift in the voltage dependence of activation. Symbols represent the mean ± SD. (c) Normalized activation curves (*I*/*I*_max_) for mHCN2 recorded at 15°C in control conditions (WT) and in the presence of 100 µM cAMP. In contrast to mHCN1, cAMP produced a clear depolarizing shift in the voltage dependence of activation, consistent with enhanced channel opening at less negative membrane potentials. Symbols represent the mean ± SD. Sample sizes are indicated in the panels; *n* denotes the number of individual quality‐controlled recorded cells included in each analysis. Exact *P*‐values for the statistical comparisons are provided in Table A3.

To determine how cyclic AMP modulates HCN channel gating across temperatures, an additional 1118 recordings were obtained from mouse HCN‐expressing cells, of which 652 passed predefined quality‐control criteria and were included in the analysis. To define an appropriate concentration for modulation experiments, intracellular cAMP concentrations ranging from 10 nM to 10 mM were tested. These experiments showed progressive acceleration of activation kinetics up to the 10–100 µM range, beyond which no substantial additional effect was observed (Figure A3a). Because 100 µM cAMP produced near‐maximal modulation and has been used in previous studies of HCN channels (Zolles et al., 2009), this concentration was selected for subsequent experiments.

**FIGURE 3:**
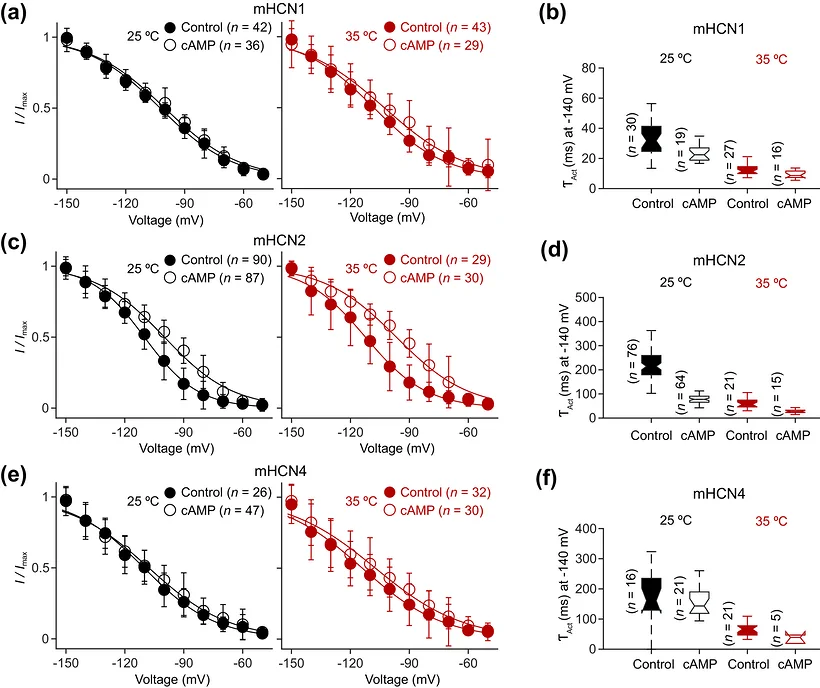
Temperature‐dependent modulation of mouse HCN channel kinetics by intracellular cAMP (100 µM). (a, c, e) Normalized activation curves (*I*/*I*_max_–*V*) for mHCN1 (a), mHCN2 (c) and mHCN4 (e) recorded in the absence (control) and presence of 100 µM intracellular cAMP at 25 (left panels) and 35°C (right panels). cAMP produced a depolarizing shift in the voltage dependence of activation, indicating that channels activate at less negative membrane potentials in the presence of cyclic nucleotide. For mHCN1, *V*_½_ shifted from −102.47 to −100.13 mV at 25°C (*P* = 0.00287) and from −108.13 to −102.82 mV at 35°C (*P* = 0.000104). For mHCN2, *V*_½_ shifted from −110.00 to −99.82 mV at 25°C (*P* < 0.0001) and from −112.36 to −96.63 mV at 35°C (*P* < 0.0001). For mHCN4, *V*_½_ shifted from −110.26 to −107.80 mV at 25°C (*P* = 0.308) and from −113.94 to −107.26 mV at 35°C (*P* = 0.0197). Symbols represent the mean ± SD. (b, d, f) Summary of activation time constants (τ_Act_) measured at −140 mV for mHCN1 (b), mHCN2 (d) and mHCN4 (f) in control conditions and in the presence of 100 µM intracellular cAMP at 25 and 35°C. For mHCN1, the median τ_Act_ decreased from 31.72 to 22.39 ms at 25°C (*P* = 0.0392) and from 11.42 to 8.68 ms at 35°C (*P* = 0.00342). For mHCN2, the median τ_Act_ decreased from 214.24 to 77.58 ms at 25°C (*P* < 0.0001) and from 56.46 to 27.59 ms at 35°C (*P* < 0.0001). For mHCN4, the median τ_Act_ decreased from 160.80 to 143.35 ms at 25°C (*P* = 0.182) and from 66.41 to 38.87 ms at 35°C (*P* = 0.00764). Box plots show the median and interquartile range; notches indicate the approximate confidence interval around the median, and whiskers extend to the most extreme data points within 1.5 times the interquartile range. Sample sizes are indicated in the panels; *n* denotes the number of individual quality‐controlled recorded cells included in each analysis. Exact *P*‐values for the statistical comparisons are provided in Table A3.

Using 100 µM intracellular cAMP, we examined its effect on voltage dependence and activation kinetics of mouse HCN channels at 25 and 35°C using the same analytical approach described for the temperature‐dependent characterization (Figure 2). Analysis of normalized current–voltage relationships revealed a depolarizing shift in the activation curves in the presence of cAMP (Figure 3a,c,e). At 35°C, *V*_½_ shifted from −108.13 to −102.82 mV for mHCN1 (shift: 5.32 mV, *P* = 0.000104), from −112.36 to −96.63 mV for mHCN2 (shift: 15.73 mV, *P* < 0.0001) and from −113.94 to −107.26 mV for mHCN4 (shift: 6.68 mV, *P* = 0.0197), indicating that cAMP facilitated channel activation at less negative membrane potentials. At 15°C, cAMP‐dependent depolarizing shifts were also observed for mHCN1, with *V*_½_ shifting from −102.36 to −100.83 mV (shift: 1.53 mV, *P* = 0.0161), and for mHCN2, with *V*_½_ shifting from −110.91 to −103.65 mV (shift: 7.26 mV, *P* < 0.0001; Figure A3b,c).

The magnitude of cAMP modulation exhibited temperature dependence and isoform‐specific differences. For mHCN1, the effect was smaller at 15 and 25°C, whereby *V*_½_ shifted from −102.47 to −100.13 mV at 25°C (shift: 2.35 mV, *P* = 0.00287), but became more pronounced at 35°C. In contrast, mHCN2 displayed robust modulation at all temperatures tested, with the strongest effect observed at 35°C; *V*_½_ shifted from −110.00 to −99.82 mV at 25°C (shift: 10.18 mV, *P* < 0.0001) and from −112.36 to −96.63 mV at 35°C (shift: 15.73 mV, *P* < 0.0001). For mHCN4, the effect of cAMP was detectable at 35°C, whereas at 25°C the shift in activation was small and not significant, with *V*_½_ changing from −110.26 to −107.80 mV (shift: 2.46 mV, *P* = 0.308). At 15°C, the currents recorded for mHCN4 were too small to meet quality‐control criteria and were therefore excluded from further analysis.

Activation kinetics were further quantified by measuring activation time constants (τ_Act_) at −140 mV (Figure 3b,d,f). At 25°C, intracellular cAMP produced a modest but significant acceleration of mHCN1 activation, reducing the median τ_Act_ from 31.72 (*n* = 30) to 22.39 ms (*n* = 19; *P* = 0.0392). In contrast, cAMP produced a much larger acceleration of mHCN2 activation, reducing the median τ_Act_ from 214.24 (*n* = 76) to 77.58 ms (*n* = 64; *P* < 0.0001). For mHCN4 at 25°C, the median τ_Act_ decreased from 160.80 (*n* = 16) to 143.35 ms (*n* = 21), but this effect was not statistically significant (*P* = 0.182).

At 35°C, cAMP produced a clearer acceleration of activation kinetics across all three functional isoforms. For mHCN1, the median τ_Act_ decreased from 11.42 (*n* = 27) to 8.68 ms (*n* = 16; *P* = 0.00342). For mHCN2, the median τ_Act_ decreased from 56.46 (*n* = 21) to 27.59 ms (*n* = 15; *P* < 0.0001). For mHCN4, the median τ_Act_ decreased from 66.41 (*n* = 21) to 38.87 ms (*n* = 5; *P* = 0.00764; Figure 3b,d,f).

Together, these results demonstrate that cAMP modulates mouse HCN channel gating in an isoform‐ and temperature‐dependent manner both by shifting the voltage dependence of activation towards more depolarized potentials and by accelerating activation kinetics. mHCN2 exhibited the strongest and most consistent sensitivity to cAMP, showing significant depolarizing shifts in *V*_½_ and marked acceleration of τ_Act_ at all temperatures tested. mHCN1 also showed significant cAMP‐dependent depolarizing shifts at all temperatures, although the effect was smaller at 15 and 25°C and became more pronounced at 35°C; acceleration of activation kinetics was likewise modest at 25°C and clearer at 35°C. In contrast, mHCN4 displayed limited modulation at 25°C, with no significant change in *V*_½_ or τ_Act_ but showed significant effects on both parameters at 35°C, although the τ_Act_ comparison at this temperature was based on a relatively small number of cAMP‐treated cells. Importantly, cAMP modulation preserved the intrinsic kinetic hierarchy of the HCN family (HCN1 fastest, HCN4 slowest), indicating that cyclic nucleotide binding enhances channel activation without altering the fundamental gating characteristics of each isoform.

### 3.4 ZD7288 inhibits mouse HCN currents in a concentration‐dependent manner across temperatures

**FIGURE A4:**
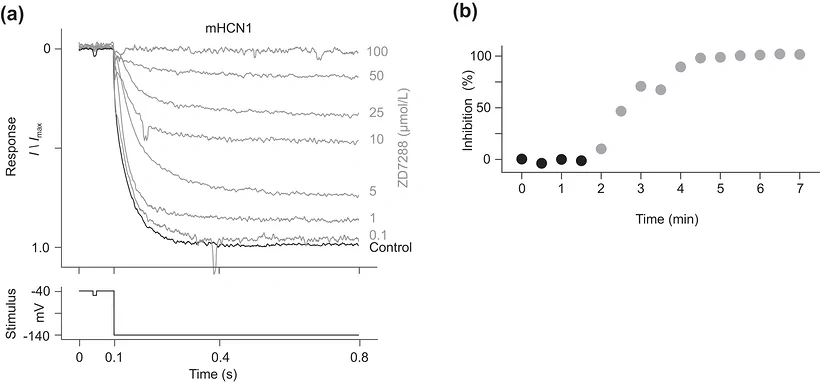
Modulation of mouse HCN channel kinetics by ZD7288. (a) Representative current traces from mHCN1‐expressing cells illustrating progressive inhibition of HCN currents with increasing concentrations of ZD7288 (from 0.1 to 100 µM) during hyperpolarizing voltage steps to −140 mV. Currents are normalized to maximal current (*I*/*I*_max_) to illustrate concentration‐dependent channel inhibition. (b) Time course of ZD7288‐mediated inhibition of HCN currents showing the gradual development of block following compound application. Inhibition was quantified over time as the percentage reduction of current amplitude relative to control.

ZD7288 is a widely used pharmacological inhibitor of HCN channels and has been used extensively to investigate the physiological role of the hyperpolarization‐activated current (*I*_h_) in both cardiac and neuronal systems (BoSmith et al., 1993; Marshall et al., 1993; Robinson & Siegelbaum, 2003; Wu et al., 2025). To characterize the temperature‐dependent inhibitory effects of ZD7288 on mouse HCN channels, channel inhibition was quantified for mHCN1, mHCN2 and mHCN4 at 25 and 35°C by measuring the HCN current evoked by a 700 ms hyperpolarizing voltage step to −140 mV using the automated patch‐clamp platform (Figure A4a). To investigate the temperature‐dependent inhibition of HCN channel gating by ZD7288, an additional 916 cells expressing mouse HCN channels were recorded, of which 490 recordings passed the predefined quality‐control criteria and were included in the analysis.

**FIGURE 4:**
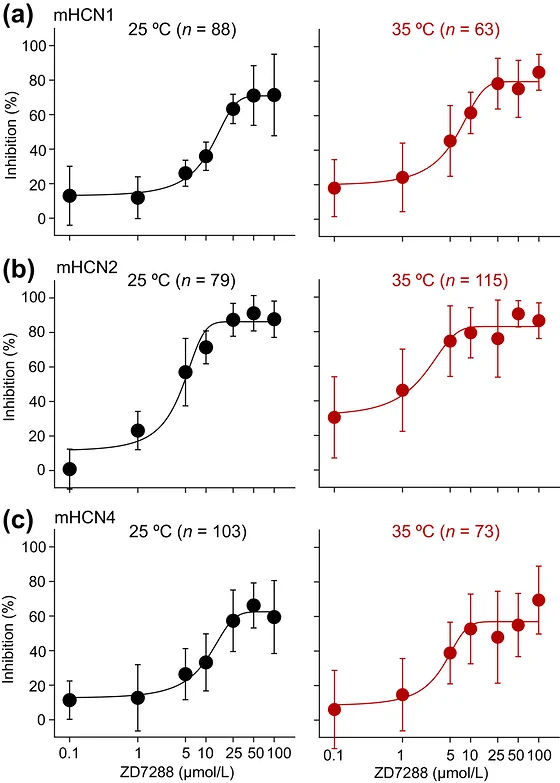
Temperature‐dependent inhibition of mouse HCN channels by ZD7288. (a–c) Concentration–response relationships for inhibition of mHCN1 (a), mHCN2 (b) and mHCN4 (c) currents by ZD7288 measured at 25 (black) and 35°C (red). Channel inhibition was quantified by measuring HCN currents evoked by a 700 ms hyperpolarizing voltage step to −140 mV using the automated patch‐clamp platform. Increasing concentrations of ZD7288 produced a progressive reduction in HCN current amplitude for all three isoforms, demonstrating dose‐dependent channel inhibition at both temperatures. The number of analysed cells is indicated in the panels. Symbols represent the mean ± SD. The extent of inhibition differed across isoforms. mHCN2 exhibited near‐complete inhibition, whereas maximal inhibition reached ∼80% for mHCN1 and 60% for mHCN4. Across all channels and temperatures, inhibition approached maximal levels at ∼50 µM ZD7288, suggesting that this concentration is sufficient to achieve near‐maximal pharmacological block of mouse HCN channels in the experimental conditions used. Sample sizes are indicated in the panels; *n* denotes the number of individual quality‐controlled recorded cells included in each analysis.

Application of ZD7288 produced concentration‐dependent inhibition of HCN channel currents across all isoforms. Representative traces illustrating progressive current reduction with increasing ZD7288 concentrations are shown for mHCN1 (Figure A4b). Consistent inhibition profiles were observed for mHCN1, mHCN2 and mHCN4, with channel activity decreasing progressively as ZD7288 concentration increased and approaching maximal inhibition at higher concentrations (Figure 4). This dose‐dependent inhibition was observed at both temperatures examined.

The extent of inhibition differed across isoforms. ZD7288 produced near‐complete inhibition of mHCN2, whereas maximal inhibition reached ∼80% for mHCN1 and 60% for mHCN4, indicating that inhibition was strongest for mHCN2 and weakest for mHCN4 in the present assay conditions (Figure 4). Across all channels and temperatures, inhibition approached maximal levels at ∼50 µM, suggesting that this concentration is sufficient to achieve near‐maximal pharmacological block of mouse HCN channels in the experimental conditions used. Time‐course analysis further demonstrated that channel inhibition developed progressively following ZD7288 application and stabilized within ∼3–4 min (Figure A4b).

Together, these results show that ZD7288 inhibits mouse HCN channel activity in a concentration‐dependent manner across temperatures. Among the functional mouse isoforms examined here, inhibition was most pronounced for mHCN2 and least pronounced for mHCN4. In the conditions used here, 50 µM ZD7288 provided near‐maximal inhibition across the tested HCN isoforms, making it an effective concentration for pharmacological suppression of HCN currents both at room temperature and in near‐physiological conditions.

### 3.5 Comparative kinetic characterization of HCN channels across species

To determine whether the kinetic properties of HCN channels are conserved across species, we recorded an additional 3240 cells expressing rat and human HCN channels, of which 1029 recordings passed the predefined quality‐control criteria and were included in the analysis.

The electrophysiological features of HCN1, HCN2, HCN3 and HCN4 channels from rats and humans were recorded in the same experimental conditions used for mouse HCN channels. Similar to mouse HCN3, rat and human HCN3 channels were also electrically silent at 15, 25 and 35°C.

**FIGURE 5:**
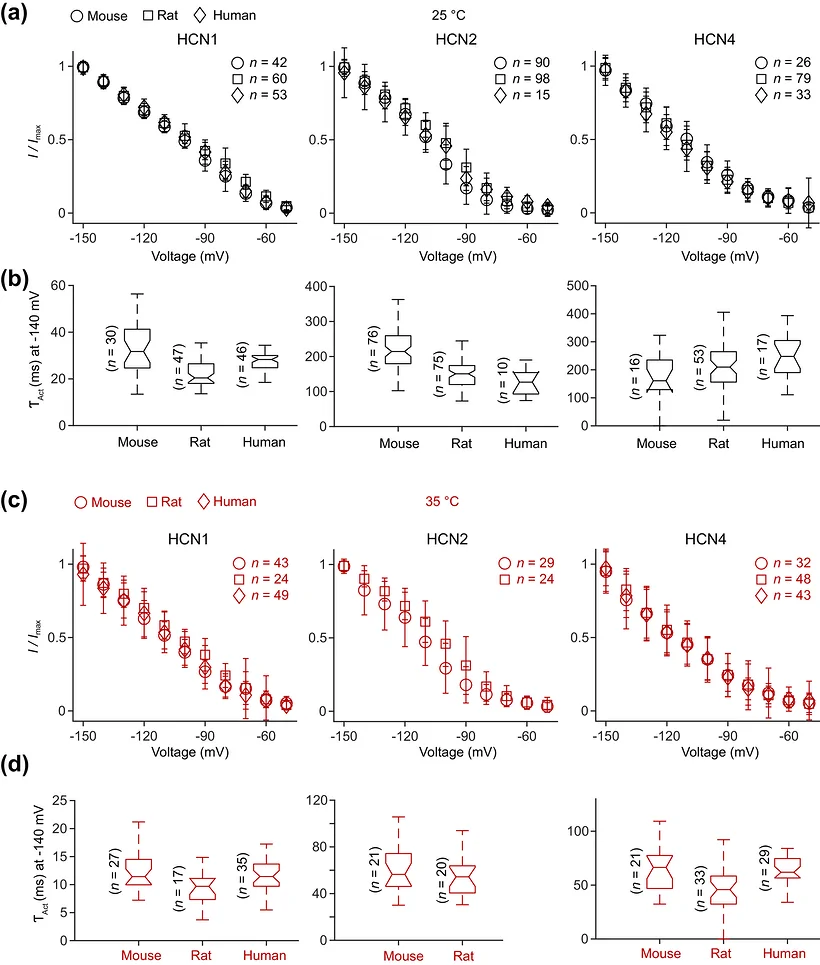
HCN channel kinetics across species (mouse, rat and human). (a, c) Normalized activation curves (*I*/*I*_max_–*V*) for HCN1, HCN2 and HCN4 channels from the mouse, rat and human, recorded at 25 (a) and 35°C (c). The voltage dependence of activation was broadly conserved across species, although modest species‐dependent differences were observed for selected isoforms. At 25°C, mouse HCN1 and HCN2 activated at more hyperpolarized potentials than the corresponding rat orthologues (HCN1, *P* < 0.0001; HCN2, *P* < 0.0001), whereas no significant species differences were observed for HCN4 relative to mouse (mouse vs. rat, *P* = 0.236; mouse vs. human, *P* = 0.107). At 35°C, rat HCN1 and rat HCN2 remained shifted to more depolarized potentials relative to mouse (HCN1, *P* = 0.00166; HCN2, *P* = 0.00193), whereas HCN4 activation remained similar across species (mouse vs. rat, *P* = 0.662; mouse vs. human, *P* = 0.928). Human HCN2 at 35°C was not included because too few recordings passed the predefined quality‐control criteria. Symbols represent the mean ± SD. (b, d) Summary of activation time constants (τ_Act_) measured at −140 mV for HCN1, HCN2 and HCN4 orthologues from mouse, rat and human recorded at 25 (b) and 35°C (d). At 25°C, rat HCN1 and rat HCN2 activated faster than the corresponding mouse orthologues (HCN1, *P* < 0.0001; HCN2, *P* < 0.0001), and human HCN2 also activated faster than mouse HCN2 (*P* = 0.0000305). At 35°C, rat HCN1 remained faster than mouse HCN1 (*P* = 0.00802), whereas mouse and rat HCN2 were not significantly different (*P* = 0.382). For HCN4, rat HCN4 activated faster than mouse HCN4 at 35°C (*P* = 0.000616), whereas human HCN4 was not significantly different from mouse (*P* = 0.768). Box plots show the median and interquartile range; notches indicate the approximate confidence interval around the median, and whiskers extend to the most extreme data points within 1.5 times the interquartile range. Sample sizes are indicated in the panels; *n* denotes the number of individual quality‐controlled recorded cells included in each analysis. Exact *P*‐values for the statistical comparisons are provided in Table A3.

**FIGURE A5:**
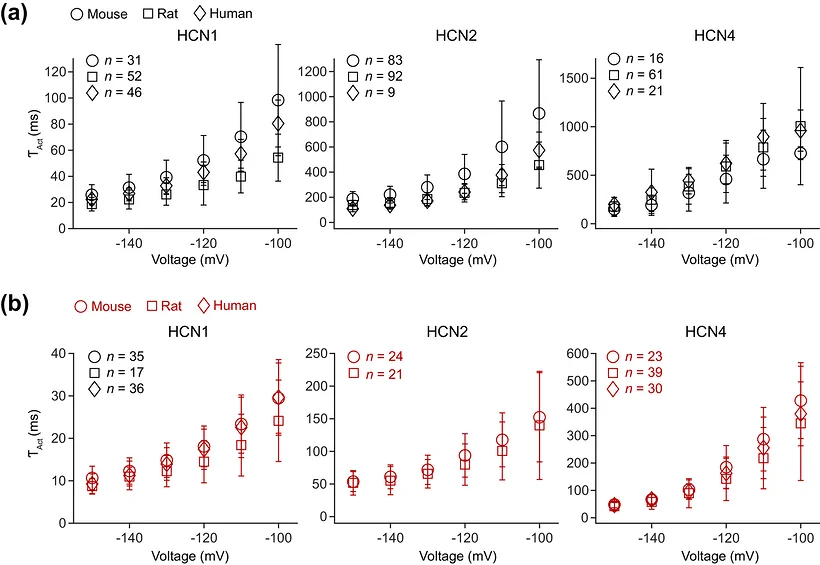
HCN channel kinetics across species (mouse, rat and human). (a) Activation time constants (τ_Act_) for mouse, rat and human HCN1, HCN2 and HCN4 channels at 25°C plotted as a function of voltage. (b) Activation time constants (τ_Act_) for HCN1, HCN2 and HCN4 channels at 35°C plotted as a function of voltage. Owing to insufficient human HCN2 recordings passing quality‐control criteria at this temperature, only mouse and rat datasets are shown for this isoform. Symbols represent the mean ± SD. Numbers next to each dataset indicate the number of cells included in the analysis after quality control filtering. Mouse, rat and human HCN3 channels were electrically silent in all tested conditions (15, 25 and 35°C) and are therefore not shown.

Activation properties were analysed using normalized current–voltage relationships (*I*/*I*_max_–*V*) and activation rise time constants (τ_Act_). The voltage dependence of activation is summarized in Figure 5a,c, and a summary comparison of activation kinetics at −140 mV is shown in Figure 5b,d. The full voltage dependence of activation time constants across species is shown separately in Figure A5.

At 25°C, the voltage dependence of activation was broadly conserved across species, although modest species‐dependent differences were observed. For HCN1, *V*_½_ was −101.48 mV in mouse, −97.58 mV in rat and −99.92 mV in human. Relative to mouse, the activation curve was shifted to more depolarized potentials in rat (*P* < 0.0001) and human channels (*P* = 0.00116). For HCN2, *V*_½_ was −109.16 mV in mouse, −103.26 mV in rat and −106.43 mV in human channels. Relative to mouse, rat HCN2 was shifted to more depolarized potentials (*P* < 0.0001), whereas the difference between mouse and human was smaller (*P* = 0.0234). For HCN4, *V*_½_ was −108.00 mV in mouse, −111.11 mV in rat and −113.60 mV in human channels, with no significant differences between mouse and rat (*P* = 0.236) or between mouse and human (*P* = 0.107; Figure 5a).

At 35°C, the overall voltage dependence of activation also remained broadly similar across species. For HCN1, *V*_½_ was −107.07 mV in mouse, −101.54 mV in rat and −104.43 mV in human channels. Relative to mouse, rat HCN1 remained shifted to more depolarized potentials (*P* = 0.00166), whereas the difference between mouse and human was not significant (*P* = 0.0562). For HCN2, *V*_½_ was −111.99 mV in mouse and −102.60 mV in rat channels, corresponding to a depolarizing shift of ∼9.39 mV in rat relative to mouse (*P* = 0.00193). Human HCN2 recordings at 35°C were insufficient for reliable inclusion in the comparison. For HCN4, *V*_½_ was −113.94 mV in mouse, −111.88 mV in rat and −113.28 mV in human channels, with no significant differences between mouse and rat (*P* = 0.662) or between mouse and human (*P* = 0.928; Figure 5c).

Activation kinetics at −140 mV were also broadly comparable across species, although selected differences were observed (Figure 5b,d). At 25°C, the median τ_Act_ for HCN1 was 31.72 ms in mouse, 20.40 ms in rat, and 28.30 ms in human channels. Rat HCN1 activated faster than mouse HCN1 (*P* < 0.0001), whereas the difference between mouse and human was not significant (*P* = 0.0751). For HCN2, the median τ_Act_ was 214.24 ms in mouse, 150.53 ms in rat and 126.83 ms in human channels. Both rat and human HCN2 activated faster than mouse HCN2 (rat, *P* < 0.0001; human, *P* = 0.0000305). For HCN4, the median τ_Act_ was 160.80 ms in mouse, 209.74 ms in rat and 247.76 ms in human channels. These differences were modest, and only the comparison between mouse and human reached nominal significance (*P* = 0.0496), whereas the difference between mouse and rat was not significant (*P* = 0.116; Figure 5b). The full τ_Act_–*V* relationships at 25°C are shown in Figure A5a.

At 35°C, the median τ_Act_ for HCN1 was 11.42 ms in mouse, 9.69 ms in rat and 11.45 ms in human channels. Rat HCN1 remained faster than mouse HCN1 (*P* = 0.00802), whereas the difference between mouse and human was not significant (*P* = 0.670). For HCN2, the median τ_Act_ was 56.46 ms in mouse and 54.34 ms in rat channels, with no significant difference between these orthologues (*P* = 0.382). Human HCN2 recordings at 35°C were insufficient for meaningful comparison. For HCN4, the median τ_Act_ was 66.41 ms in mouse, 45.80 ms in rat and 61.95 ms in human channels. Rat HCN4 activated faster than mouse HCN4 (*P* = 0.000616), whereas the difference between mouse and human was not significant (*P* = 0.768; Figure 5d). The corresponding full τ_Act_–*V* relationships at 35°C are shown in Figure A5b.

Overall, these results indicate that the major features of HCN channel gating are broadly conserved across species, although modest species‐dependent differences are evident for selected isoforms and temperatures. Rat HCN1 and HCN2 showed faster activation than the corresponding mouse orthologues at 25°C, and rat HCN1 remained faster at 35°C. In contrast, HCN4 gating was more similar across species, with only limited differences in voltage dependence and activation kinetics. Human HCN1 and HCN4 generally resembled the corresponding mouse or rat orthologues, whereas human HCN2 could be evaluated reliably only at 25°C.

### 3.6 Electrophysiological data availability for HCN channels

All electrophysiological recordings generated in this study have been made publicly available through the Channelpedia (Ranjan et al., 2011) platform to facilitate data sharing, reproducibility and further analysis by the scientific community. The dataset includes raw electrophysiological traces, processed kinetic parameters and associated experimental metadata for the HCN channel experiments reported in this study.

The recordings are provided in a standardized HDF5‐based file format (The HDF Group, 2025), enabling efficient storage and cross‐platform accessibility of large electrophysiological datasets. Through the Channelpedia interface, users can browse individual recordings, visualize electrophysiological responses and download complete datasets corresponding to specific channels, species, temperatures or experimental conditions.

To facilitate programmatic access and downstream data analysis further, we provide dedicated application programming interfaces (APIs) for both MATLAB and Python. These APIs allow users to retrieve and analyse electrophysiological recordings directly from Channelpedia within commonly used scientific computing environments. Example scripts and documentation are available to guide users in accessing and processing the data.

Together, these resources provide an open and standardized framework for accessing large‐scale electrophysiological datasets, enabling researchers to reproduce the analyses presented in this study and to perform independent investigations of HCN channel kinetics.

## 4 DISCUSSION

HCN channels are key regulators of membrane excitability in both cardiac and neuronal tissues, where they generate the pacemaker currents *I*_f_ and *I*_h_ (DiFrancesco, 1995; DiFrancesco & Ojeda, 1980; Pape, 1996). Although HCN channels have been studied extensively, many previous investigations have focused on individual isoforms or selected experimental conditions, often using different expression systems, temperatures and voltage protocols. In the present study, we performed a systematic electrophysiological characterization of mammalian HCN channels across isoforms, species, temperatures and modulatory conditions using a standardized automated patch‐clamp workflow. This approach enabled direct comparison of channel properties in controlled experimental conditions while generating a large, publicly accessible electrophysiological dataset.

To maintain standardization in kinetic profiling and allow comparison with our previous Kv channel studies, we applied six voltage protocols interrogating activation, deactivation, inactivation, ramp responses, action potential waveforms and inactivation recovery. Given the distinct biophysical properties of HCN channels, however, not all protocols were equally informative. Because HCN channels open upon hyperpolarization, activate relatively slowly and lack classical inactivation, the most informative analyses in the present study were derived primarily from the activation and deactivation protocols. At the same time, given that all raw and processed electrophysiological data have been made publicly available, the dataset can support more fine‐grained analyses of selected protocols, specific channel states or individual experimental conditions that were beyond the scope of the present study.

A notable observation from our analysis is that HCN3 did not produce detectable currents when expressed as a homomeric channel in heterologous systems, despite confirmed gene and protein expression and membrane localization (data not shown). This finding was consistent across species (mouse, rat and human), temperatures (15, 25 and 35°C) and expression systems (CHO and HEK cells), suggesting that HCN3 might require additional regulatory factors, heteromeric assembly with other HCN isoforms or specific cellular environments to produce functional currents. This observation also provides a possible explanation for why HCN3 remains the least studied member of the HCN family compared with HCN1, HCN2 and HCN4. There are ∼120 publications on HCN3 to date, compared with ∼800 for each of the other HCN isoforms. At the same time, previous studies have reported conflicting observations regarding HCN3 channel activity, indicating that its functional properties might depend strongly on cellular context or regulatory mechanisms (Hassinen et al., 2017; Stieber et al., 2005). Our results, therefore, do not exclude physiological roles for HCN3 in native tissues, but they do indicate that in the standardized heterologous conditions used here, HCN3 does not produce detectable homomeric currents.

Among the remaining functional isoforms, HCN1 exhibited the fastest activation kinetics, whereas HCN4 was generally the slowest, with HCN2 showing intermediate behaviour in conditions where the kinetic separation was most clearly resolved. Importantly, this canonical ordering was most evident at 35°C. At 25°C, the distinction between HCN2 and HCN4 was less clear, and in some conditions, HCN2 activation was comparable to or even slower than HCN4 (Table 1). Thus, our data indicate that the commonly described HCN1–HCN2–HCN4 kinetic hierarchy is most clearly observed at physiological temperatures. These findings are broadly consistent with previous studies reporting rapid activation of HCN1 and slower gating of HCN4, reflecting their distinct physiological roles in neuronal signalling and cardiac pacemaking (Chen et al., 2001; Robinson & Siegelbaum, 2003).

**TABLE 1:** Qualitative summary of temperature effects on mouse HCN channels.

Isoform	Detectable current	Activation kinetics (25 → 35 °C)	Deactivation kinetics (25 → 35°C)	Shift in activation *V*_½_	*Q*_10_ of activation	Overall temperature sensitivity
mHCN1	+++	+++	+++	++	++	++
mHCN2	+++	+++	+++	0	+++	+++
mHCN3	0	NA	NA	NA	NA	NA
mHCN4	+++	++	++	0 to +	+	++

Symbol key: +++, strong effect; ++, moderate effect; +, weak effect; 0, no clear or non‐significant effect; NA, not applicable.

Although the strongest activation in our standardized protocols was observed at relatively negative membrane potentials, this does not imply that HCN channels are physiologically irrelevant near typical subthreshold voltages. For example, for mHCN1 at 25°C in control conditions, the normalized activation curve shown in Figure 2b and the expanded view inFigure A2c indicate that ∼10%–20% of the maximal current is available within the physiological subthreshold range of −85 to −65 mV. Although activation is relatively slow, such current might still contribute to resting membrane behaviour, slow subthreshold oscillations, recovery following inhibitory input and rebound depolarization. Moreover, relatively small shifts in voltage dependence or kinetics driven by temperature or cyclic nucleotide modulation might substantially alter channel availability within this voltage range. Thus, the physiological impact of HCN channels cannot be inferred solely from maximal current amplitude at strongly hyperpolarized potentials but should be considered in the context of native membrane voltages and modulatory state.

Temperature strongly influenced HCN channel gating in our experiments. Increasing temperature markedly accelerated both activation and deactivation kinetics across all isoforms, consistent with the thermodynamic sensitivity of conformational transitions underlying channel opening. In addition, modest shifts in the voltage dependence of activation were observed for HCN1 and HCN4 at higher temperatures. These results emphasize the importance of conducting electrophysiological measurements at physiologically relevant temperatures, because kinetic parameters obtained at room temperature might underestimate channel activation rates in vivo. The strong temperature dependence observed here is consistent with previous reports and highlights the dynamic nature of HCN channel gating (Wu et al., 2025).

Although 15°C is below typical mammalian physiological temperature and was not the main focus of the present study, these recordings still extend the thermal range of the dataset and provide useful constraints for quantitative analyses of temperature‐dependent HCN gating. In addition, because the major features of HCN channel kinetics were broadly conserved across isoforms and species, the low‐temperature data might be valuable for comparative physiology, for modelling excitable tissues in altered thermal conditions and for interpreting situations in which cold exposure or impaired thermal regulation affects membrane excitability.

Consistent with the established regulatory role of cyclic nucleotides, intracellular cAMP shifted activation to more depolarized potentials and accelerated activation kinetics across the functional HCN isoforms. The magnitude of this effect depended on both isoform and temperature, with mHCN2 showing the strongest modulation and mHCN1 the weakest. These observations support the view that cyclic nucleotide binding to the cyclic nucleotide‐binding domain stabilizes channel conformations that favour opening, thereby facilitating activation. Our standardized analysis provides a directly comparable quantification of this modulatory effect across HCN isoforms and temperatures. Screening across a wide range of cAMP concentrations indicated that much of the modulatory effect was already observed at 10–100 µM, suggesting that 10 µM cAMP might have been sufficient to capture near‐maximal effects in this assay. Because CHO cells express endogenous phosphodiesterase (PDE) transcripts relevant to cAMP turnover, including *Pde4a*, *Pde4b* and *Pde7a*, this result is unlikely to arise from a complete lack of cAMP‐degrading machinery in the host cells (Table A4). However, because PDE activity and local cAMP compartmentation were not measured directly, and comparable data from native tissues are not available here, the apparent cAMP sensitivity should be interpreted as assay context dependent. Nevertheless, we used 100 µM cAMP in the detailed modulation experiments because this concentration was used in previous studies and provided a robust, standardized condition across isoforms. An additional point emerging from these analyses is that the influence of temperature on HCN kinetics was particularly strong (Table 2). Although cAMP consistently accelerated activation, the effect of a 10°C rise in temperature was generally larger than the effect of 100 µM cAMP. This comparison highlights that thermal conditions exert a major influence on HCN channel gating and should always be considered when interpreting modulatory or pharmacological effects. Thus, even canonical regulation by cyclic nucleotides should be understood in the broader context of the prevailing temperature.

**TABLE 2:** Qualitative summary of modulation of mouse HCN channels by cAMP and ZD7288.

Isoform	cAMP effect on *V*_½_	cAMP effect on τ_Act_	Temperature dependence of cAMP effect	ZD7288 inhibition	Relative sensitivity to ZD7288
mHCN1	++	+ to ++	Stronger at 35°C	++	Intermediate
mHCN2	+++	+++	Strong at all temperatures, greatest at 35°C	+++	Highest
mHCN3	NA	NA	NA	NA	NA
mHCN4	0 at 25°C; ++ at 35 °C	0 at 25°C; ++ at 35°C	Clearer at 35°C	+	Lowest

Symbol key: +++, strong effect; ++, moderate effect; +, weak effect; 0, no clear or non‐significant effect; NA, not applicable.

In addition to physiological modulation, we examined the pharmacological inhibition of HCN channels by ZD7288, a widely used HCN blocker. ZD7288 produced concentration‐dependent inhibition of HCN1, HCN2 and HCN4 at both 25 and 35°C, with mHCN2 showing the greatest sensitivity and mHCN4 the weakest inhibition in the tested conditions. Across isoforms and temperatures, inhibition approached a plateau by ∼50 µM, indicating that this concentration is sufficient to achieve near‐maximal suppression of mouse HCN currents in the present assay (Table 2). These data provide a useful quantitative reference for the pharmacological modulation of HCN channels in standardized conditions. At the same time, because ZD7288 is frequently used in native tissues expressing mixed HCN populations, the isoform dependence of inhibition should be considered when interpreting pharmacological experiments.

Comparative analysis across mouse, rat and human orthologues indicated that major features of HCN channel gating are broadly conserved across species (Table 3). Both the voltage dependence of activation and the general isoform‐specific kinetic patterns were maintained across orthologues in standardized conditions, suggesting that core structural determinants of HCN gating are evolutionarily conserved. Nevertheless, some technical limitations should be noted. Human HCN2 currents could not be recorded reliably despite repeated attempts and were therefore not included in the species comparison. In addition, mHCN4 currents at 15°C were too small and slow to satisfy quality‐control criteria and were excluded from detailed analysis at that temperature. These exclusions reflect the practical constraints of standardized high‐throughput electrophysiology and should be taken into account when interpreting completeness across all channel–condition combinations. Even so, the overall conservation observed across the successfully recorded orthologues supports the continued use of rodent models for investigating HCN channel physiology while also providing a reference framework for assessing species‐dependent differences.

**TABLE 3:** Qualitative summary of cross‐species comparison of HCN channels.

Isoform	Detectable current across species	Conservation of *V*_½_	Conservation of activation kinetics	Main species‐dependent trend
HCN1	+++	++	++	Rat activates faster than mouse; human broadly similar to mouse
HCN2	+++ (human limited at 35°C)	++	++	Rat and human faster than mouse at 25°C; rat shifted to more depolarized potentials at 35°C
HCN3	0	NA	NA	Electrically silent across mouse, rat and human
HCN4	+++	+++	++ to +++	Broadly similar across species with only modest differences

Symbol key: +++, strong conservation or clear effect; ++, moderate conservation/difference; +, weak effect; 0, no detectable current; NA, not applicable.

The present dataset might also be relevant to understanding how temperature‐sensitive ion‐channel properties influence neural function in disease. Recent work has emphasized that altered thermal conditions and temperature extremes can challenge neural resilience and might aggravate susceptibility to neurological dysfunction, particularly in disorders of excitability (Alonso & Marder, 2020). In parallel, computational studies of temperature compensation in rhythmic circuits have shown that neural systems can preserve function across changing temperatures by reorganizing the relative contributions of underlying currents and that the effects of perturbations might differ qualitatively across temperatures. In this context, the standardized HCN dataset presented here provides an experimentally grounded reference for how temperature alters channel gating across mammalian isoforms and species. Such information might be useful not only for electrophysiological interpretation and ion‐channel modelling, but also for understanding how thermal conditions could reshape subthreshold excitability, pacemaking and circuit stability in both physiological and pathological settings (Gulcebi et al., 2025).

An important aspect of this work is the public availability of the electrophysiological dataset generated in this study. All raw recordings and processed data have been deposited in the Channelpedia platform in a standardized file format together with MATLAB and Python APIs that enable programmatic access. The availability of such large‐scale electrophysiological datasets should facilitate reproducibility, enable independent reanalysis and support the development of quantitative models of ion channel function. Because the present study emphasized standardized comparisons across many experimental conditions, the released dataset also provides an opportunity for future analyses focused on individual isoforms, specific voltages, rare gating behaviours or protocol‐specific features that were outside the scope of this work.

## 5 CONCLUSION

In summary, our results provide a comprehensive and standardized characterization of HCN channel kinetics across isoforms, species, temperatures and modulatory conditions. The strong temperature dependence of gating, the isoform‐ and temperature‐dependent effects of cyclic nucleotide modulation and the concentration‐dependent inhibition by ZD7288 together highlight key regulatory features of HCN channel function. More broadly, these data establish a comparative physiological framework for interpreting HCN channel behaviour in controlled experimental conditions. Together with the publicly accessible dataset, this work provides a reusable resource for future experimental, pharmacological and computational studies of HCN channels.

## AUTHOR CONTRIBUTIONS

Rajnish Ranjan: Conceptualization, Data curation, Formal analysis, Investigation, Methodology, Project administration, Supervision, Validation, Visualization, Writing—original draft, Writing—review & editing; Emmanuelle Logette: Conceptualization, Investigation, Methodology, Supervision, Validation; Mirjia Herzog: Investigation; Valerie Buchillier: Investigation; Enrico Scantamburlo: Software, Data curation; Henry Markram: Conceptualization, Funding acquisition. All authors approved the final version of the manuscript and agree to be accountable for all aspects of the work in ensuring that questions related to the accuracy or integrity of any part of the work are appropriately investigated and resolved. All persons designated as authors qualify for authorship, and all those who qualify for authorship are listed.

## CONFLICT OF INTEREST

The authors declare no competing interests.

## GENERATIVE AI STATEMENT

During the preparation and revision of this manuscript, the authors used ChatGPT (OpenAI) for editorial assistance to improve clarity, structural syntax, and the narrative flow. No original experimental data, data analysis, statistical analysis, figures, or scientific interpretations were generated by artificial intelligence. All AI‐assisted content was critically reviewed, verified, and edited by the authors, who take full responsibility for the accuracy, integrity, and final content of this manuscript.

## Data Availability

The raw electrophysiological recordings and analysis code are available via Channelpedia (https://www.channelpedia.net/). Any additional data or materials required to reanalyse the findings of this study are available from the corresponding author upon reasonable request. This study did not generate new, unique reagents.

## 1

**TABLE A1:** Sequence description of the HCN channel genes used in the study. Nucleotide variations with respect to the original RefSeq are listed.

Gene	Protein	NCBI Ref Seq	Nucleotide modifications compared to RefSeq
Mouse HCN genes			
*HCN1*	Hyperpolarization‐activated cyclic nucleotide‐gated channel 1 (HCN1)	NM_010408	
*HCN2*	Hyperpolarization‐activated cyclic nucleotide‐gated channel 2 (HCN2)	NM_008226	1 silent mutation nt12C>A
*HCN3*	Hyperpolarization‐activated cyclic nucleotide‐gated channel 3 (HCN3)	NM_008227	1 silent mutation nt15 A vs G
*HCN4*	Hyperpolarization‐activated cyclic nucleotide‐gated channel 4 (HCN4)	NM_001081192	
Rat HCN genes			
*HCN1*	Hyperpolarization‐activated cyclic nucleotide‐gated channel 1 (HCN1)	NM_053375	
*HCN2*	Hyperpolarization‐activated cyclic nucleotide‐gated channel 2 (HCN2)	NM_053684	nt 2431 CCT corrected in TCT + 2 silent: nt 2103 G vs. A, nt 306 T vs. C
*HCN3*	Hyperpolarization‐activated cyclic nucleotide‐gated channel 3 (HCN3)	NM_053685	
*HCN4*	Hyperpolarization‐activated cyclic nucleotide‐gated channel 4 (HCN4)	NM_021658	
Human HCN genes			
*HCN1*	Hyperpolarization‐activated cyclic nucleotide‐gated channel 1 (HCN1)	NM_021072	
*HCN2*	Hyperpolarization‐activated cyclic nucleotide‐gated channel 2 (HCN2)	NM_001194	
*HCN3*	Hyperpolarization‐activated cyclic nucleotide‐gated channel 3 (HCN3)	NM_020897	nt1763CAG>CGG
*HCN4*	Hyperpolarization‐activated cyclic nucleotide‐gated channel 4 (HCN4)	NM_005477	

*Note*: Synonymous modifications introduced in the cloning primers to improve primer quality are labelled as ‘modified nt’.

**TABLE A2:** List of primers used for PCR amplification and cloning in pENTR/TOPO vector of rat, mouse and human HCN genes.

Gene	Accession no.	Forward primer (5′–3′)	Reverse primer (5′–3′)
Mouse HCN genes			
*HCN1*	NM_010408	CACCATGGAAGGCGGCGGCAAAC	TCATAAATTCGAAGCAAAACGGG
*HCN2*	NM_008226	CACCATGGATGCGCGAGGGGGC	TCACAAGTTGGAAGAGAGGC
*HCN3*	NM_008227	CACCATGGAGGAGGAGGCGCGGCC	TCACATGTTGGCAGAAATTTGGGGAC
*HCN4*	NM_001081192	F1‐GCCGCCCCCTTCACCATGGACAAGCTGCCGCCGT	R1‐CAGGCTGGAAGACCTCGAAACGCAGCTTGGTCAG
		F2‐AGGTCTTCCAGCCTGGGGATTACATCATCCGCGA	R2‐GGCGCGCCCACCCTTTCATAAATTAGACGGCAGT
Rat HCN genes			
*HCN1*	NM_053375	CACCATGGAAGGCGGCGGCAAG	TCATAAATTCGAAGCAAAACGTGG
*HCN2*	NM_053684	CACCCAGCCCCGCGGCCTCTGCGCGCCCGGCCAGCAG	CTGCTGGCCGGGCGCGCAGAGGCCGCGGGGCTGGGTG
*HCN3*	NM_053685	CACCATGGAGGAGGAGGCGCGGCC	TCACATGTTGGCAGAAATTTGGGGAC
*HCN4*	NM_021658	F1‐GCCGCCCCCTTCACCATGGACAAGCTGCCGCCGT	R1‐CAGGCTGGAAGACCTCGAAACGCAGCTTGGTCAG
		F2‐AGGTCTTCCAGCCTGGGGATTACATCATCCGCGA	R2‐GGCGCGCCCACCCTTTCATAAATTAGACGGCAGT
Human HCN genes			
*HCN1*	NM_021072	F1‐GCCGCCCCCTTCACCATGGAAGGAGGCGGCAAGC	R1‐GCCTTGGTATCTGTGTTCATAGTAATCATG
		F2‐CACAGATACCAAGGCAAAATCTTTGATGAGG	R2‐GGCGCGCCCACCCTTTCATAAATTTGAAGCAAATCG
*HCN2*	NM_001194		
*HCN3*	NM_020897	CACCATGGAGGCAGAGCAGCGG	TTACATGTTGGCAGAAAGCT
*HCN4*	NM_005477	F1‐GCCGCCCCCTTCACCATGGACAAGCTGCCGCCGT	R1‐CAGGCTGGAAGACCTCGAAACGCAGCTTGGTCAG
		F2‐GAGGTCTTCCAGCCTGGGGACTACATCATCCGGG	R2‐GGCGCGCCCACCCTTTCATAGATTGGATGGCAG

**TABLE A3:** Exact *P*‐values for the statistical tests used to support the conclusions in Figures 1, 2, 3, 4, 5 and Figure A3.

Figure	Comparison	Test	*P*‐value	Exact *P*‐value
Figure 1d	Global comparison across CHO‐FT, mHCN1, mHCN2, mHCN3 and mHCN4	Kruskal–Wallis test	<0.0001	1.23 × 10^−50^
Figure 1d	CHO‐FT vs. mHCN3	Dunn–Sidak *post hoc* test	0.115	0.115
Figure 1d	mHCN1 vs. mHCN3	Dunn–Sidak *post hoc* test	0.000547	0.000547
Figure 1d	mHCN2 vs. mHCN3	Dunn–Sidak *post hoc* test	<0.0001	<1.2 × 10^−50^
Figure 1d	mHCN4 vs. mHCN3	Dunn–Sidak *post hoc* test	<0.0001	5.96 × 10^−6^
Figure 2b	mHCN1 *V*_½_, 25 vs. 35°C	Mann–Whitney *U*‐test	<0.0001	1.37 × 10^−6^
Figure 2b	mHCN2 *V*_½_, 25 vs. 35°C	Mann–Whitney *U*‐test	0.118	0.118
Figure 2b	mHCN4 *V*_½_, 25 vs. 35°C	Mann–Whitney *U*‐test	0.2139	0.2139
Figure 2e	mHCN1 τ_Act_, 25 vs. 35°C	Two‐sample *t*‐test	<0.0001	1.35 × 10^−10^
Figure 2e	mHCN2 τ_Act_, 25 vs. 35°C	Two‐sample *t*‐test	<0.0001	3.09 × 10^−34^
Figure 2e	mHCN4 τ_Act_, 25 vs. 35°C	Two‐sample *t*‐test	<0.0001	5.23 × 10^−5^
Figure 3a	mHCN1 25°C *V*_½_, control vs. cAMP	Mann–Whitney *U*‐test	0.002	0.002865
Figure 3a	mHCN1 35°C *V*_½_, control vs. cAMP	Mann–Whitney *U*‐test	0.0001	0.0001041
Figure 3c	mHCN2 25°C *V*_½_, control vs. cAMP	Mann–Whitney *U*‐test	<0.0001	1.22 × 10^−18^
Figure 3c	mHCN2 35°C *V*_½_, control vs. cAMP	Mann–Whitney *U*‐test	<0.0001	5.46 × 10^−8^
Figure 3e	mHCN4 25°C *V*_½_, control vs. cAMP	Mann–Whitney *U*‐test	0.308	0.308
Figure 3e	mHCN4 35°C *V*_½_, control vs. cAMP	Mann–Whitney *U*‐test	0.01974	0.01974
Figure 3b	mHCN1 25°C τ_Act_, control vs. cAMP	Mann–Whitney *U*‐test	0.0392	0.0392
Figure 3b	mHCN1 35°C τ_Act_, control vs. cAMP	Mann–Whitney *U*‐test	0.003	0.00342
Figure 3d	mHCN2 25°C τ_Act_, control vs. cAMP	Mann–Whitney *U*‐test	<0.0001	1.05 × 10^−23^
Figure 3d	mHCN2 35°C τ_Act_, control vs. cAMP	Mann–Whitney *U*‐test	<0.0001	1.10 × 10^−5^
Figure 3f	mHCN4 25°C τ_Act_, control vs. cAMP	Mann–Whitney *U*‐test	0.182	0.182
Figure 3f	mHCN4 35°C τ_Act_, control vs. cAMP	Mann–Whitney *U*‐test	0.007	0.007643
Figure A3b	mHCN1 15°C *V*_½_, control vs. cAMP	Mann–Whitney *U*‐test	0.0161	0.0161
Figure A3c	mHCN2 15°C *V*_½_, control vs. cAMP	Mann–Whitney *U*‐test	<0.0001	4.42 × 10^−13^
Figure 5a	*V*_½_ mouse HCN1 vs. rat HCN1 at 25°C	Mann–Whitney *U*‐test	<0.0001	1.16 × 10^−7^
Figure 5a	*V*_½_ mouse HCN1 vs. human HCN1 at 25°C	Mann–Whitney *U*‐test	0.001	0.00116
Figure 5a	*V*_½_ mouse HCN2 vs. rat HCN2 at 25°C	Mann–Whitney *U*‐test	<0.0001	1.50 × 10^−10^
Figure 5a	*V*_½_ mouse HCN2 vs. human HCN2 at 25°C	Mann–Whitney *U*‐test	0.023	0.02342
Figure 5a	*V*_½_ mouse HCN4 vs. rat HCN4 at 25°C	Mann–Whitney *U*‐test	0.236	0.2364
Figure 5a	*V*_½_ mouse HCN4 vs. human HCN4 at 25°C	Mann–Whitney *U*‐test	0.107	0.1072
Figure 5c	*V*_½_ mouse HCN1 vs. rat HCN1 at 35°C	Mann–Whitney *U*‐test	0.001	0.001661
Figure 5c	*V*_½_ mouse HCN1 vs. human HCN1 at 35°C	Mann–Whitney *U*‐test	0.056	0.0562
Figure 5c	*V*_½_ mouse HCN2 vs. rat HCN2 at 35°C	Mann–Whitney *U*‐test	0.001	0.001934
Figure 5c	*V*_½_ mouse HCN2 vs. human HCN2 at 35°C	NA	NA	NA
Figure 5c	*V*_½_ mouse HCN4 vs. rat HCN4 at 35°C	Mann–Whitney *U*‐test	0.662	0.6621
Figure 5c	*V*_½_ mouse HCN4 vs. human HCN4 at 35°C	Mann–Whitney *U*‐test	0.927	0.9275
Figure 5b	τ at −140 mV mouse HCN1 vs. rat HCN1 at 25°C	Mann–Whitney *U*‐test	<0.0001	4.52 × 10^−5^
Figure 5b	τ at −140 mV mouse HCN1 vs. human HCN1 at 25°C	Mann–Whitney *U*‐test	0.075	0.07508
Figure 5b	τ at −140 mV mouse HCN2 vs. rat HCN2 at 25°C	Mann–Whitney *U*‐test	<0.0001	1.69 × 10^−12^
Figure 5b	τ at −140 mV mouse HCN2 vs. human HCN2 at 25°C	Mann–Whitney *U*‐test	<0.0001	3.05 × 10^−5^
Figure 5b	τ at −140 mV mouse HCN4 vs. rat HCN4 at 25°C	Mann–Whitney *U*‐test	0.116	0.1162
Figure 5b	τ at −140 mV mouse HCN4 vs. human HCN4 at 25°C	Mann–Whitney *U*‐test	0.050	0.04962
Figure 5d	τ at −140 mV mouse HCN1 vs. rat HCN1 at 35°C	Mann–Whitney *U*‐test	0.008	0.008017
Figure 5d	τ at −140 mV mouse HCN1 vs. human HCN1 at 35°C	Mann–Whitney *U*‐test	0.670	0.6702
Figure 5d	τ at −140 mV mouse HCN2 vs. rat HCN2 at 35°C	Mann–Whitney *U*‐test	0.382	0.3823
Figure 5d	τ at −140 mV mouse HCN2 vs. human HCN2 at 35°C	NA	NA	NA
Figure 5d	τ at −140 mV mouse HCN4 vs. rat HCN4 at 35°C	Mann–Whitney *U*‐test	0.0006	0.0006159
Figure 5d	τ at −140 mV mouse HCN4 vs. human HCN4 at 35°C	Mann–Whitney *U*‐test	0.768	0.7681

*Note*: Comparisons were performed using the statistical tests indicated in the table. Where *P* < 0.0001, values are reported as <0.0001 in accordance with journal guidelines. For non‐significant comparisons, the exact *P*‐values are also provided. NA indicates that the comparison was not performed because insufficient recordings passed the predefined quality‐control criteria.

**TABLE A4:** Expression profiles of PDE‐related genes in CHO, HEK and CV1 cells.

Gene group	Gene symbol	Gene ID	CHOWT	HEKWT	CV1WT
PDE genes					
	*Pde6d*	100762580	67.01	35.7	50.99
	*Pde5a*	100755315	16.7	4.16	4.08
	*Pde12*	100769630	11.86	6.39	5.22
	*Pde10a*	100751551	6.03	1.06	0.78
	*Pde4b*	100774886	4.25	1.25	0.8
	*Pde4a*	100759696	3.43	0.9	0.8
	*Pde7a*	100760222	3.13	8.19	0.51
	*Pde1b*	100689193	0.98	1.1	0.61
	*Pde2a*	100750420	0.38	0	8.02
	*Spdef*	100756355	0.33	0	0
	*Pde6g*	100764811	0.32	0	0
	*Pde1c*	100774969	0.26	0	27.4
	*Pde6b*	100766007	0.09	0	0
	*Pde4d*	100754362	0.04	9.77	17.84
	*Pde6a*	100762641	0	0	0.31
	*Pde1a*	100762910	0	1.13	0
	*Pde4c*	100770327	0	0	0
	*Pde7b*	100753277	0	0	7.16
	*Pde11a*	100756511	0	0.23	0
	*Pde8b*	100755939	0	5.55	0.32
	*Pde3a*	100774584	0	0	0
	*Pde8a*	100759944	0	5.11	1.81
	*Pde9a*	100765436	0	0.48	0
	*Pde3b*	100775064	0	7.92	0.33
Adenylyl cyclases					
	*Adcy7*	100756132	17.35	1.51	4.88
	*Adck2*	100771195	14.98	0	1.24
	*Pradc1*	100770278	14.82	0	1.07
	*Adck5*	100762357	13.61	1.41	1.01
	*Adcy6*	100768368	12.81	6.95	25.75
	*Adck1*	100759124	4.26	2.21	2.51
	*Adcy9*	100751372	4.14	5.05	2.7
	*Cdadc1*	100763867	3.41	3.83	1.16
	*Adcy3*	100770498	1.73	20.84	8.89
	*Madcam1*	100750395	0.05	0	0
	*Adcy10*	100767272	0	0	0
	*Adcy1*	100764561	0	6.72	0
	*Adcy2*	100773811	0	0	1.42
	*Adcy4*	100768998	0	0	0
	*Adcy5*	100765958	0	0.52	1.74
	*Adcyap1r1*	100772260	0	1.8	0
cAMP effector					
PKA subunits					
	*Prkar1a*	100773792	183.15	87.14	345.2
	*Prkaca*	100768997	70.3	79.43	70.34
	*Prkag1*	100765754	33.02	25.52	26.74
	*Prkab1*	100755226	30.94	9.76	2.92
	*Prkar2b*	100766449	27.92	46.16	48
	*Prkacb*	100767097	18.08	1.89	1.89
	*Prkaa1*	100769574	11.01	41.8	23.23
	*Prkab2*	100772359	8.08	6.35	1.94
	*Prkag2*	100758649	7.53	10.13	111.76
	*Prkar1b*	100770073	0.1	0	0
	*Prkaa2*	100767909	0	21.97	20.19
	*Prkag3*	100770459	0	0	0
EPAC genes					
	*Rapgef1*	100764804	37.47	13.33	19.97
	*Rapgef2*	100754995	26.73	15.69	8.18
	*Rapgef3*	100767619	7.59	0.73	1.35
	*Rapgef6*	100763755	5.46	6.42	3.67
	*Rapgef4*	100774346	1.22	7.98	22
	*Rapgefl1*	100774035	0.71	0.38	0
	*Rapgef5*	100769866	0	4.57	12.7
HCN genes					
	*Hcn3*	100772247	0.18	4.46	3.72
	*Hcn2*	100751563	0	13.92	26.06
	*Hcn1*	100753109	0	0	2.63
	*Hcn4*	100758754	0	0.89	0.99
cAMP extrusion					
	*Abcc5*	100774121	59.4	20.41	21.82
	*Abcc4*	100766754	29.39	6.03	22.6
	*Abcc1*	100689208	19.04	1.37	1.52
	*Abcc3*	100767558	5.67	0.76	22.21
	*Abcc10*	100757623	2.43	2.92	8.01
	*Abcc2*	100689220	0.06	0	0.97
	*Abcc9*	100769198	0	0	7.66
	*Abcc8*	100764394	0	0.16	0
	*Abcc6*	100752070	0	0	0
cAMP compartmentation					
	*Akap12*	100772605	128.16	4.08	7.51
	*Akap1*	100760847	26.45	20.66	13.39
	*Akap7*	100757731	26	1.6	1.59
	*Akap8*	100771696	24.37	1.72	1.44
	*Akap13*	100768171	19.63	5.4	8.07
	*Akap11*	100769001	18.63	3.34	2.6
	*Akap8l*	100754234	12.77	97.35	137.44
	*Akap9*	100767118	9.54	0.79	0.64
	*Akap10*	100759380	7.41	24.73	14
	*Akap17a*	100769666	1.44	0.13	0
	*Akap3*	100773279	0.8	0	0
	*Akap5*	100768039	0	0	0
	*Akap6*	100756287	0	1.12	0.34
Housekeeping genes					
	*Actb*	100689477	2656.72	2526.34	9886.05
	*Gapdh*	100736557	5338.95	1938.97	1398.37
	*Hprt1*	100769768	84.07	312.92	87.41
	*Pgk1*	100689467	438.61	1005.82	838.77
	*Polr2a*	100689016	41.22	92.1	42.74
	*Rplp0*	100756201	2899.28	3766.36	5098.02
	*Rpl13a*	100773637	1190.52	96.4	58.6
	*Eef1a1*	100689276	5874.28	20048.95	15429.63
	*Ppia*	100769490	472.98	1438.71	739.35
	*Tbp*	100751579	17.29	27.39	22.45

*Note*: Data are expressed as fragments per kilobase of transcript per million mapped reads (FPKM) and were obtained from the study by Ranjan et al. (2019).
